# Feasibility of Care Coordination to Reduce Unnecessary Hospitalization For Assisted Living Residents

**DOI:** 10.1093/geroni/igaf122.4120

**Published:** 2025-12-31

**Authors:** Ann Reddy, Laura Dionne, Grace Wittenberg, Peter Serina, Nate Hunkins, Martha Etzell, Aimee VanDenberghe, Ellen McCreedy

**Affiliations:** Brown University, Providence, Rhode Island, United States; Brown University, Providence, Rhode Island, United States; Brown University, Providence, Rhode Island, United States; Salem Hospital, Salem, Massachusetts, United States; Bluestone Physician Services, Stillwater, Minnesota, United States; Bluestone Physician Services, Stillwater, Minnesota, United States; Bluestone Physician Services, Stillwater, Minnesota, United States; Brown University, School of Public Health, Providence, Rhode Island, United States

## Abstract

Almost half of Assisted Living Community (ALC) residents visit the emergency department (ED) yearly, experiencing more visits and longer stays compared to community-dwelling older adults. People living with Alzheimer’s disease and related dementias (ADRD) are at greater risk for delirium, falls, and accelerated decline associated with increased ED visits. Each transition provides an opportunity for care coordination and avoidance of unnecessary hospital admission. Bluestone Accountable Care Organization developed ED Early Response, a care coordination program. Care managers provide timely, structured information to ED providers via phone and fax within 120 minutes of ED registration. We assessed the feasibility of, and adherence to, the program. Between November 2023 and June 2024, we enrolled 1,376 patients with 1,989 eligible ED visits (mean: 1.4 visits per patient), 1,237 ED visits for patients with ADRD and 752 ED visits for patients without ADRD. Qualifying visits occurred during working hours (8 a.m. - 4 p.m.), with 82.5% of visits (n = 1,641) identified via electronic admission, discharge, and transfer notifications, and 17.5% of visits (n = 348) identified through direct communication between ALCs and care managers. Care managers successfully provided real-time information to ED providers for 44% of the eligible ED visits (547 of 1,237) for patients with ADRD, and for 40% of visits (304 of 752) for patients without ADRD. Care managers self-reported a hospital avoidance rate of 11%. While adherence was lower than anticipated, early structured communication could reduce unnecessary hospital admissions for ALC residents.

